# Rates and Correlates of Incident Type 2 Diabetes Mellitus Among Persons Living With HIV-1 Infection

**DOI:** 10.3389/fendo.2020.555401

**Published:** 2020-11-23

**Authors:** Yuanfan Ye, Sadeep Shrestha, Greer Burkholder, Anju Bansal, Nathaniel Erdmann, Howard Wiener, Jianming Tang

**Affiliations:** ^1^ Department of Epidemiology, University of Alabama at Birmingham, Birmingham, AL, United States; ^2^ Department of Medicine, University of Alabama at Birmingham, Birmingham, AL, United States

**Keywords:** HIV-1 infection, obesity, statin, type 2 diabetes mellitus, race

## Abstract

The prevalence of various comorbidities continue to rise in aging persons living with HIV-1 infection (PLWH), and our study here aimed to assess the rates and correlates of incident type 2 diabetes mellitus (T2DM) in PLWH from a retrospective, southeastern U.S. cohort. Based on electronic health records, we examined patient demographics, body mass index (BMI), HIV-1-related outcomes, hepatitis C virus co-infection, common comorbidities (e.g. shingles and asthma), usage of protease inhibitors, and usage of statins as potential correlates for T2DM occurrence. Among 3,975 PLWH with ≥12 months of follow-up between January 1999 and March 2018, the overall rate of incident T2DM was 135 per 10,000 person-years, almost 2-fold higher than the rate reported for the general U.S. population. In multivariable models (354 T2DM patients and 3,617 control subjects), sex, BMI, nadir CD4^+^ T-cell count, HIV-1 viral load (VL) and duration of statin use were independent correlates of incident T2DM (adjusted *P* <0.05 for all), with clear consistency in several sensitivity analyses. The strongest associations (adjusted odds ratio/OR >2.0 and *P* <0.0001) were noted for: i) statin use for ≥6 months (OR = 10.2), ii) BMI ≥30 kg/m^2^ (OR = 3.4), and iii) plasma VL ≥200 copies/ml (OR = 2.2). Their collective predictive value was substantial: the C-statistic for area under the receiver operating characteristics curve was 0.87 (95% CI = 0.84-0.91), showing close similarity between two major racial groups (C-statistic = 0.87 for African Americans and 0.91 for European Americans). Overall, these findings not only establish a promising algorithm for predicting incident T2DM in PLWH but also suggest that patients who are obese and use statins should require special consideration for T2DM diagnosis and prevention.

## Introduction

In persons living with human immunodeficiency virus type 1 (HIV-1) infection (PLWH), adherence to combination antiretroviral therapy (cART) can effectively suppress viral replication and drive plasma viral load (VL) to an undetectable level, followed by partial immune reconstitution and a close-to-normal life expectancy (1). While mortality from AIDS-defining conditions has dramatically declined in the cART era, the burden of chronic, non-AIDS comorbidities continues to rise in the aging PLWH population (2). For example, the prevalence of diabetes is known to be much higher among PLWH than the general population (3), but longitudinal data on incident type 2 diabetes mellitus (T2DM) in PLWH are lacking.

According to recent estimates from the American Diabetes Association (ADA), about 1.5 million new diabetes cases are diagnosed every year in the United States (U.S.) general population (4), and there is abundant evidence that age, obesity, race, and genetic factors contribute to T2DM (5–7). These factors are also known to influence T2DM in PLWH, but additional factors, including chronic inflammation and long-term cART use, and exposure to various HIV-1 protease inhibitors can alter the risk for and complicate the management of T2DM and other metabolic disorders among PLWH (8–11). Our analyses of retrospective data from a southeastern U.S. clinical cohort were intended to gain new insights into key factors associated with incident T2DM in the contemporary PLWH population. 

## Subjects and Methods

### Study Cohort

PLWH attending an outpatient HIV clinic (The 1917 Clinic) at University of Alabama at Birmingham (UAB) (12) were enrolled and continuously followed between January 1999 and March 2018 for routine care and monitoring of coinfections and comorbidities. For this sub-study, diagnosis of T2DM followed the new ADA recommendations based on a combination of any two tests of hemoglobin A1c (≥42 mmol/mol), fasting plasma glucose (≥126 mg/dl), and random plasma glucose test (≥200 mg/dl) (13–15). In most cases, medication records (e.g., prescription of metformin) matched T2DM diagnosis, and a total of 354 incident T2DM cases and 3,621 control subjects were identified based on the following exclusion criteria (**Figure S1 in Supplemental Materials**): i) unknown or ambiguous self-reported races, ii) less than 18 years old at the time of enrollment, iii) receiving organ transplantation (systemic immunosuppression other than HIV-1 infection), iv) less than 12-month active follow-up, v) diagnoses of T2DM before enrollment (prevalent cases, *n* = 159), vi) confirmed type 1 diabetes mellitus, vii) missing two critical, HIV-1-related variables (CD4^+^ T-cell counts and VL). All subjects provided written informed consent for participation in the study, and research protocols pertinent to this sub-study were approved by an Institutional Review Board at UAB.

### Dataset Retrieved for Various Analyses

The following variables were retrieved from a central, electronic database: i) enrollment date, ii) demographics (sex, birth date, race/ethnicity), iii) risk factors for HIV-1 acquisition (sexual orientation and injection drug use), iv) the duration of protease inhibitor use; v) the category and duration of statin use; vi) nadir CD4^+^ T-cell (CD4) count (cells/µl) in the last 2 years before the incident T2DM diagnosis (for T2DM incident cases) or the last clinical visit (for control subjects), and vii) HIV-1 viremia (VL, RNA copies/ml of plasma) in the last 2 years before the incident T2DM diagnosis (for T2DM cases) or the last clinical visit (for control subjects). 

### Descriptive Statistics

Data from the overall PLWH cohort and several stratified groups (defined by sex, race, and enrollment periods) were first analyzed for incident rates of T2DM per 10,000 person-years (PY) of follow-up, along with their 95% confidence intervals (CI). Subjects were then stratified by T2DM status (incident cases vs. controls) for direct comparison of baseline characteristics (**Table 1**), which included i) counts and percentages for each categorical variable, ii) mean and standard deviation (SD) of continuous variables, iii) median and interquartile range for CD4 and VL. Collinearity among all variables was assessed by nonparametric (Spearman) pairwise correlation analyses. To focus on robust findings, we adopted a newly proposed *P*-value threshold (*P* <0.005) for statistical significance (16). This *P*-value cut-off was intended to ensure a corresponding Bayes factor between 14 and 26, which is in favor of rejecting null hypothesis while reducing false positive rate (16).

**Table 1:** Overall characteristics of the study cohort stratified by type 2 diabetes mellitus (T2DM).

Characteristics	No T2DM (*n* = 3,621)	Incident T2DM (*n* = 354)	*P*-value^b^	*Q*
Age at enrollment (mean ± SD)	38.8 ± 10.9	44.3 ± 9.4	<0.0001	<0.001
Age group at incident T2DM or last clinical visit			<0.0001	<0.001
18–44 years	1738 (48.0)	111 (31.4)	Ref.	
45–64 years	1712 (47.3)	223 (63.0)	<0.0001	<0.001
≥65 years	171 (4.7)	20 (5.6)	0.002	0.003
Sex			<0.001	<0.001
Men	2821 (21.5)	245 (69.2)	Ref.	
Women	779 (77.9)	108 (30.5)	0.0001	<0.001
Transgenders	21 (0.6)	1 (0.3)	>0.50	>0.50
Race/ethnicity			0.49	>0.50
African American (AA)	2124 (58.7)	202 (57.1)	Ref.	
European American (EA)	1419 (39.2)	147 (41.5)	0.45	0.50
Others	78 (2.2)	5 (1.4)	0.40	0.46
BMI group at incident DM or last clinical visit (Missing 364 subjects)			<0.0001	<0.001
<25 *(kg*/m^2^)	1348 (40.6)	58 (19.9)	Ref.	
25–29 *(kg*/m^2^)	1091 (32.9)	79 (27.0)	0.003	0.005
≥30 *(kg*/m^2^)	880 (26.5)	155 (53.1)	<0.0001	<0.001
Median nadir CD4 count over the last 24 months			0.021	0.027
<200 cells/µl	850 (23.5)	81 (22.9)	0.36	0.43
200-500 cells/µl	1231 (34.0)	145 (41.0)	0.006	0.008
>500 cells/µl	1540 (42.5)	128 (36.1)	ref	
Highest VL over the last 24 months			<0.0001	<0.001
<200	2108 (58.2)	168 (47.5)	Ref.	
200-23,596 (Q3)	614 (17.0)	92 (26.0)	<0.0001	<0.001
>23,596	899 (24.8)	94 (26.5)	0.044	0.055
Protease inhibitor (PI) use			<0.001	<0.001
No PI in the last 2 years	2267 (62.6)	184 (52.0)	Ref.	
PI use for 0–2 years	435 (12.0)	54 (15.2)	0.010	0.014
PI use for ≥2 years	919 (25.4)	116 (32.8)	0.001	0.002
Statin use				<0.001
Never	2753 (76.0)	111 (31.4)	Ref.	
Atorvastatin	379 (10.5)	104 (29.4)	<0.0001	<0.001
Pravastatin	312 (8.6)	82 (23.2)	<0.0001	<0.001
Pitavastatin	2 (0.1)	0 (0.00)	0.69	0.69
Others	175 (4.8)	57 (16.0)	<0.0001	<0.001
Duration of statin use			<0.0001	<0.001
No use	2753 (76.0)	111 (31.3)	Ref.	
Use for <6 months	115 (3.2)	30 (8.5)	<0.0001	<0.001
Use ≥6 months	753 (20.8)	213 (60.2)	<0.0001	<0.001

^a^For comparisons involve multiple categories within a specific variable, the group with the largest sample size is set as the norm/reference.

### Multivariable Models to Identify Independent Correlates of Incident T2DM

A linear regression model was fitted to test multicollinearity between independent correlates before fitting them to the final multivariable model. A backward stepwise selection procedure was adopted to identify factors independently associated with T2DM (adjusted *P*-value less than 0.05), and a joint, multivariable model was used to assess the relative impact of each factor on T2DM, with adjusted odds ratios and 95% CIs as the key summary statistics. The clinical utility of these factors in predicting T2DM was assessed by the C-statistic and its 95% CI for area under the receiver operating characteristics curve (ROC) (13). These analytical procedures were performed using the statistical analysis software (SAS), version 9.3 (SAS Institute, Cary, North Carolina, USA).

### Secondary Analysis and Sensitivity Analysis

To fully assess the timing of T2DM in our study cohort, several potential correlates (e.g., sex and race) of T2DM were also evaluated in Kaplan-Meier (KM) curves and Cox-proportional hazard models, using enrollment date as the starting point. Moreover, several sensitivity analyses considered the enrollment periods as proxies for the evolving cART regimens, as well as varying guidelines for HIV-1 screening and cART initiation. The new guidelines of statin therapy, as introduced in 2013, also required separate analyses of T2DM cases diagnosed before 2013 and since 2013. Consistent findings from the final (reduced) multivariable model were also validated separately in EA and AA subgroups. Potential fluctuations in rates over the 18-year follow-up period was assessed by comparing rates among four time intervals (4.5 years each) in two major racial groups (AA and EA). The Joinpoint Trend Analysis software was used to detect possible trends, as described elsewhere (17, 18).

### Data and Resource Availability

The retrospective datasets analyzed during this study are available from the corresponding author upon reasonable request.

## Results

### Overall T2DM Rates and Disparity Between Men and Women

**Figure 1:**
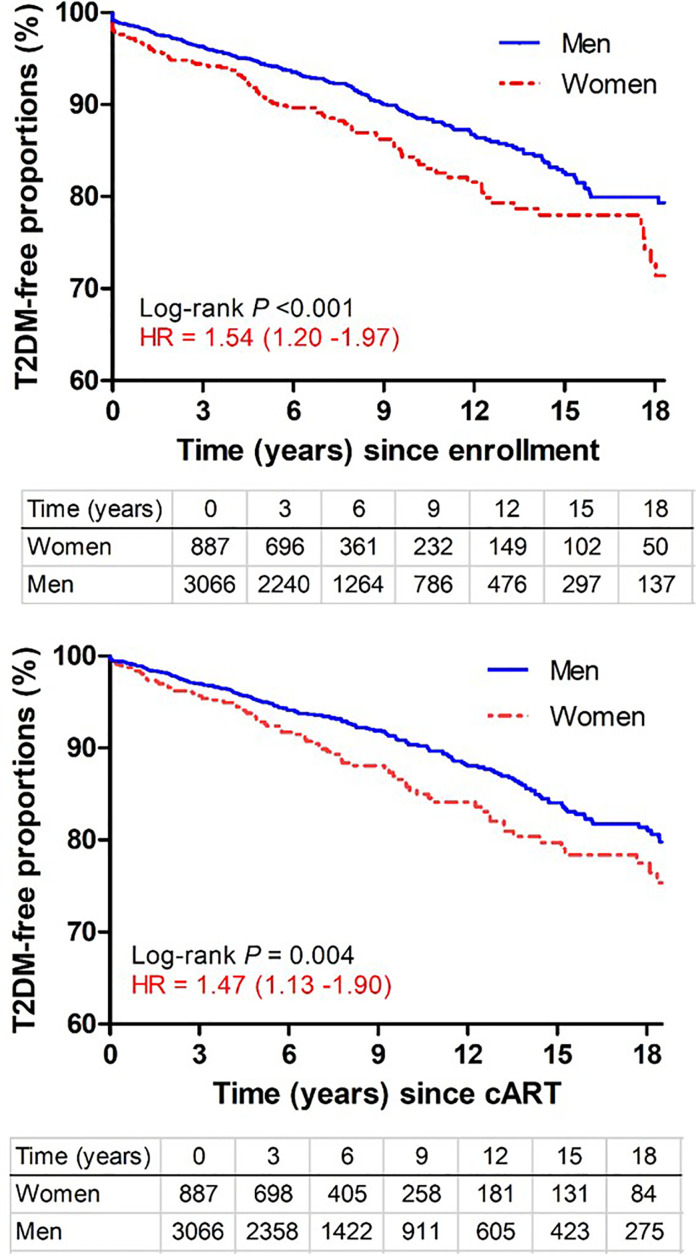
Kaplan-Meier curves showing time from study enrollment or initiation of combination antiretroviral therapy (cART) to diagnosis of type 2 diabetes mellitus (T2DM) in PLWH stratified by sex. Numbers of subjects remaining at various visit intervals (between January 1999 and March 2018) are indicated for each group (excluding 22 transgender subjects). Estimates of hazards ratios (HR) use men as the reference group.

Among 3,975 subjects included in this study (**Figure S1 in Supplemental Materials**), 354 incident T2DM cases were diagnosed after a total of 26,256 PY of follow-up, with an overall rate of 135 incidents per 10,000 PY (95% CI = 121-149 per 10,000 PY), which was almost identical to pooled data from a recent meta-analysis (137 per 10,000 PY, 95% CI = 130-200) (19) but two-fold higher than the general U.S. population (67 per 10,000 PY) (20). When several subgroups were compared, the rates were similar for AA (144 cases per 10,000 PY) and EA (123 cases per 10,000 PY) (*P* >0.50) but clearly differed between men (122 cases per 10,000 PY) and women (179 cases per 10,000 PY) (*P* <0.0001). Kaplan-Meier (KM) curves (**Figure 1**) indicated that women progressed to T2DM faster (hazards ratio = 1.54; 95% CI = 1.20-1.97) than men during the study period (*P* <0.001). Sex-specific disparity was also evident (*P* = 0.004) when all follow-up time before cART initiation was disregarded (HR = 1.47, 95% CI = 1.13-1.90 for women after cART initiation) (**Figure 1**).

### No Trend Over Time

When the 18-year study interval was divided into four equal periods (i.e., 4.5 years each), the rates of T2DM diagnosis were highly comparable for all pairwise comparisons (*P* >0.50), before and after stratification by two major racial groups (AA and EA) (**Figure S2 in Supplemental Materials**). The Joinpoint regression did not reveal any linear trend for both AA and EA (*P* > 0.20).

### Age at T2DM Onset

The median age of T2DM diagnosis was 49.4 years (IQR: 43.2-55.6) (**Figure S3 in Supplemental Materials**), about 5 years younger than reports (median age = 54 years) for the general U.S. population (21). Subjects between 45 and 65 years old were at the highest risk for incident T2DM (**Table 1**), being consistent with the general U.S. population. The median age of T2DM was similar for AA (48.7 years, IQR = 40.7-65.3) and EA (51.2 years, IQR = 45.0-65.8) (*P* = 0.31) (**Figure S3 in Supplemental Materials**).

### Screening for Factors Associated With T2DM Occurrence

As an initial screening step, univariate analyses (**Table 1**) revealed that T2DM cases and control subjects differed in age at study enrollment, age groups at the end of follow-up, sex, BMI, peak VL over the last 24 months of follow-up, cART regimens containing PI, statin use, and length of statin use (*P* <0.005 for at least one group in each variable). By contrast, the two groups were quite similar (*P* >0.05) in terms of their racial composition, while nadir CD4 count over the last 24 months differed slightly for one of these three subgroups (200–500 cells/µl, *P* = 0.008) (**Table 1**).

### Independent Correlates of T2DM Occurrence

In a reduced multivariable model (**Table 2**) applicable to 3,975 subjects (including 354 incident T2DM cases), sex, obesity, nadir CD count, HIV-1 viremia, and statin use were independently associated with the onset of T2DM (adjusted *P* <0.05 for all). Individual factors with the strongest effect (adjusted OR, aOR >2.0, *P* <0.0001) were: i) statin use for ≥6 months (aOR = 10.21), ii) BMI ≥30 kg/m^2^ (aOR = 3.36), and iii) plasma VL ≥200 copies/ml (aOR = 2.24). The effects of age, sex, and PI-containing cART regimens were attenuated when other factors were accounted for (i.e., through statistical adjustments). In multicollinearity test, there was no evidence to support multicollinearity between the key predictors in the multivariable model.

**Table 2:** Independent correlates of incident T2DM, as defined by a reduced multivariable model.

Factors in the final model^a^	n (%)	Adjusted OR^b^	95% CI^b^	*P*-value
Sex				
Men	3,063 (77.1)	Ref.	–	
Women	886 (22.3)	1.47	1.13-1.93	0.005
Transgenders	22 (0.55)	1.23	0.16-9.71	>0.50
BMI at incident DM or last visit (missing 364)				
<25 *(kg*/m^2^)	1,406 (38.9)	Ref.	–	
25–29 *(kg*/m^2^)	1,168 (32.4)	1.46	1.01-2.10	0.043
≥30 *(kg*/m^2^)	1,033 (28.7)	3.36	2.40-4.70	<0.0001
Nadir CD4^+^ T-cell count^a^ in the last 24 months				
<200 cells/µl	931 (23.4)	1.02	0.69-1.49	>0.50
200–500 cells/µl	1,374 (34.6)	1.40	1.06-1.42	0.020
>500 cells/µl	1,666 (42.0)	Ref.	–	
Highest plasma viral load^a^ in the last 24 months				
<200	2,273 (57.2)	Ref.	–	
200–23,596 (Q3)	705 (17.8)	2.24	1.64-3.05	<0.0001
>23,596	993 (25.0)	2.21	1.55-3.15	<0.0001
Duration of statin use				
No use	2,864 (72.1)	Ref	–	
Use for <6 months	145 (3.7)	8.92	5.87-13.58	<0.0001
Use ≥6 months	962 (24.3)	10.21	7.71-13.53	<0.0001

^a^At least one entry in each categorical variable has shown independent association with T2DM (adjusted P < 0.05).

^b^The odds ratio (OR) and 95% confidence interval have been adjusted for all factors in the model, as well as age, race, study enrollment dates, exposure to HIV-1 protease inhibitors, and statin groups (atorvastatin, lovastatin, and simvastatin as lipophilic; pravastatin, rosuvastatin, and fluvastatin as hydrophilic). For comparisons involve multiple categories within a specific variable, the group with the largest sample size is set as the norm/reference.

**Figure 2:**
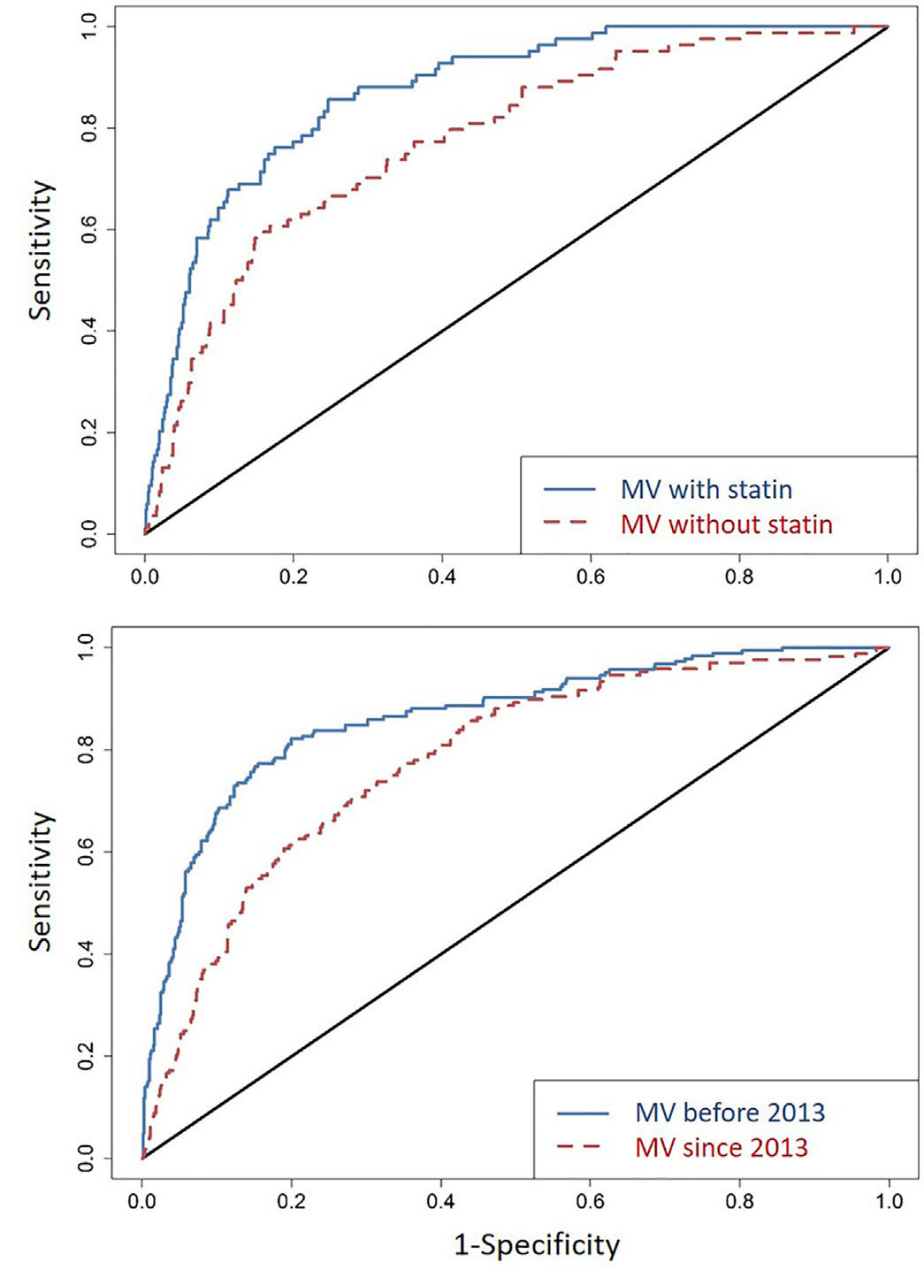
Area under the receiver operating characteristics curves (ROCs) in several rounds of comparative analyses. First (top panel), for all factors identified as independent predictor of incident T2DM in a reduced multivariable (MV) model (**Table 2**), the C-statistic for ROC (line in blue) is 0.87 (95% CI = 0.84-0.91); removing statin use from the model (line in red) leads to an ROC of 0.78 (95% CI = 0.73-0.83). Second (bottom panel), with reference to the new guidelines of statin therapy (introduced in 2013), T2DM diagnosed before 2013 (line in blue) and since 2013 (line in red) have ROC of 0.86 (95% CI = 0.83-0.90) and 0.86 (95% CI = 0.78-0.84), respectively.

When all factors independently associated with T2DM were combined, their collective predictive value was substantial: the C-statistic for ROC was 0.87 (95% CI = 0.84-0.91) (**Figure 2**), with close similarity for two major racial groups (C-statistic = 0.87 and 0.91 for AA and EA, respectively). Removing statin use from the model decreased the overall C-statistic to 0.78 (95% CI = 0.73-0.83) (**Figure 2**). In a sensitivity analysis, the C-statistics were similar between T2DM diagnosed before 2013 (when new guidelines of statin therapy were introduced) (ROC = 0.86; 95% CI = 0.83-0.90) and T2DM diagnosed since 2013 (ROC = 0.81; 95% CI = 0.78-0.84) (**Figure 2**).

### Partial Collinearity Among Factors Associated With T2DM Occurrence

In pairwise correlation analyses, Spearman |*ρ*| was <0.30 for all (**Table 3**) except for i) age group and BMI group *ρ* = 0.80, *P* <0.0001), ii) peak VL and nadir CD4 groups over the final 24 months of follow-up (*ρ* = -0.51, *P* <0.0001), and iii) statin use and age (*ρ* = 0.40, *P* <0.0001). These relationships were all well expected.

**Table 3:** Partial correlation among factors associated with T2DM^a^.

	Sex	Age	BMI	Statin use	CD4 count	HIV-1 VL
Sex	1.00	0.05	0.15	-0.02	0.03	<0.01
Age	0.001	1.00	0.80	0.40	0.07	-0.22
BMI	<0.0001	0.001	1.00	0.16	0.21	-0.21
Statin use	0.294	<0.0001	<0.0001	1.00	0.13	-0.18
CD4 count	0.053	<0.0001	<0.0001	<0.0001	1.00	-0.51
HIV-1 VL	0.862	<0.0001	<0.0001	<0.0001	<0.0001	1.00

^a^Defined by positive or negative Spearman rho (ρ) and restricted to variables shown in **Table 2**. The ρ values (two decimals) for pairwise correlation are shown on and above the diagonal; the corresponding P values (at least three decimals) are listed below the diagonal.

### Discussion

Consistent with data from a recent meta-analysis, which included 44 studies across the world (except for the Eastern Mediterranean area) (19), our findings based on a southeastern U.S. clinical cohort clearly indicate that the rates of incident T2DM have doubled in PLWH as compared with the general U.S. population. These observations appear to be generalizable, as separate analyses of two major racial groups (AA and EA) and four follow-up intervals (calendar dates) produced highly comparable summary statistics for the rates and correlates of T2DM. On the basis of a reduced multivariable model, the final estimates for ROC statistics hovered around 90% for both AA and EA subgroups, well exceeding the commonly used clinical diagnostic cut-off of 80% (22). The final algorithms for predicting incident T2DM in PLWH, which have not been attempted before, now provide promising leads for further refinements and clinical considerations.

Apart from the confirmation of several conventional risk factors (age, obesity and the length of PI-containing cART) associated with T2DM, our analyses here revealed a strong and independent impact of statin use (**Table 2**), regardless of statin categories (**Table 1**). In contrast, earlier studies only suggest a moderate risk (OR <2.0) in adults without HIV-1 infection (23).

Statins are cost-effective and widely prescribed drugs for lowering cholesterol level and preventing CVD in the U.S., especially since 2003 (24). The U.S. National Center for Health Statistics reported that more than 26% of American adults aged 40 and above used statin in 2012 (24). In previous reports, the benefits of statins outweigh all known disease risks, including T2DM or prediabetes (25, 26). A recent systematic review suggested that the reduction of LDL-C level through upregulation of LDL receptor expression by statin therapy should not introduce any adverse effects. However, if the LDL-C reduction is not related to LDL receptors, the possible drug-induced adverse effects could counterbalance the clinical benefits of statin therapy (27), suggesting that drug-drug interactions or effects through an alternative drug target are possible.

In 2013, an ACC/AHA Task Force introduced a comprehensive discussion on the initiation of statin therapy. In addition to LDL-C level and its corresponding age at test, history of diabetes and its diagnosis age, along with life style and family histories of ASCVD are now all taken into consideration before the initiation of statin therapy (28). According to the new guidelines for risk assessment, patients diagnosed with diabetes and LDL-C between 70–189 mg/dl at ages between 40 and 75 years are all recommended for moderate to high-intensity statin therapies (28). However, the 2013 ACC/AHA Blood Cholesterol Guideline did not consider the risk of T2DM after the initiation of statin therapy. Even with 2013 as a new milestone for prescribing statins, our statistical models for T2DM diagnosed before and since 2013 (**Figure 2**) did not reveal striking differences. Both models retained satisfactory fitting (ROC >0.8), even when the available follow-up period was still relatively short (about 5 years) after 2013.

PLWH are known to face high risks for CVD as a result of persistent immune activation, arterial inflammation, endothelial dysfunction, elevated coagulation, and various side effects of cART (29–31). Thus, statins have an increasing clinical application for managing maladaptive inflammatory cascades, and their usage is expected to gain further momentum in the PLWH population. If both lipophilic (atorvastatin, lovastatin, and simvastatin) and hydrophilic (pravastatin, rosuvastatin, and fluvastatin) statins indeed pose a clear risk for T2DM in PLWH, even during a short period of exposure (e.g., <6 months) (**Table 2**), follow-up studies may need to closely monitor the changes in glycated hemoglobin levels (32) and develop a concurrent strategy for minimizing the risk of T2DM. For example, high-dose vitamin D supplementation has shown convincing benefits for insulin sensitivity in pre-diabetic subjects (33, 34), PLWH with vitamin D deficiency and insufficiency may require timely vitamin D supplementation.

Another observation that seems to distinguish PLWH from the general population is the modest disparity in T2DM incidence between male and female PLWH (**Figure 1**). The difference is readily detected in both cross-sectional and longitudinal data, before and after statistical adjustments for other prominent factors (**Table 2**). In the general U.S. population, rates of incident T2DM are comparable for men and women, but differences in rates of pre-diabetes are discernible (20). The underlying biology is still elusive.

When HIV-1-specific variables are considered, CD4 T-cell count and viremia also show modest impact on T2DM onset (**Table 2**). As both can fluctuate over time with and without cART, our focus was on the last 24 months of follow-up when most PLWH were under effective treatment. The occasional viremia (viral blips), defined by plasma VL ≥200 RNA copies/ml, is well expected, and at least one other longitudinal study (a French HIV-1 cohort) has reported that detectable VL is associated with a modest risk for incident T2DM when compared to non-detectable VL (35). In the new era that calls for prompt HIV-1 diagnosis and linkage to care (the 90:90:90 initiative) (36), at least 27% of PLWH will continue to be viremic and thus at high risk for T2DM. 

One major limitation of this study is the lack of family history for study participants. T2DM tends to run in families, mostly through shared genetic and environmental factors, as well as similarity in lifestyle (37). Some of these gaps can be closed for PLWH with active follow-up, and the addition of high-throughput genotyping, especially the use of fine-mapping platforms, should facilitate the analyses of “causal variants” previously established in the general population (38). Genomic and epigenomic datasets can also help define the underlying biological pathways for T2DM (39) or enhance predictive algorithms (40). These frontiers will remain as active research areas to further benefit precision medicine.

Overall, adequate sample size and sufficient follow-up in our study cohort have provided a valuable opportunity for illustrating the complexity of T2DM risks pertinent to contemporary PLWH population. Although replication of key findings cannot be done for additional cohorts right away, multiple sensitivity analyses and stratifications by sex, race, and various study intervals have provided promising assurance that the statistical models are robust enough to be clinically relevant. In particular, appropriate interventions for statin users are expected to bear the bulk of immediate impact on the T2DM landscape in PLWH populations.

## Data Availability Statement

The raw data supporting the conclusions of this article will be made available by the authors, without undue reservation.

## Ethics Statement

The studies involving human participants were reviewed and approved by Institutional Review Board at University of Alabama at Birmingham. All subjects provided written informed consent for participation in the study.

## Author Contributions

JT, SS, AB, and NE designed the study. YY and GB managed the datasets. YY, HW, and JT analyzed the data. YY and JT wrote the manuscript. All authors contributed to the article and approved the submitted version.

## Funding

This study was supported in part by the United States government, through two R21 awards to JT (AG051309 and AI134282) from the National Institutes of Health. Datasets from the UAB 1917 Clinic Cohort were made possible through Center for AIDS Research (P30 AI027767) and the CNICS Network (R24 AI067039), both of which are funded by the National Institute of Allergy and Infectious Diseases (NIAID) and the National Heart, Lung, and Blood Institute (NHLBI).

## Conflict of Interest

The authors declare that the research was conducted in the absence of any commercial or financial relationships that could be construed as a potential conflict of interest.
