# Supplementary material for: Rates and Correlates of Incident Type 2 Diabetes Mellitus Among Persons Living With HIV-1 Infection
Source: Front Endocrinol (Lausanne). 2020 Nov 23;11:555401. doi: 10.3389/fendo.2020.555401 (PMC7719801; doi:10.3389/fendo.2020.555401)

Supplemental Materials

Figure S1. **PLWH with and without incident type 2 diabetes mellitus (T2DM) between January 1999 and March 2018 in a clinical cohort.** Among 4,846 PLWH with follow-up visits, 354 T2DM cases and 3,621 control subjects are selected for analyses.

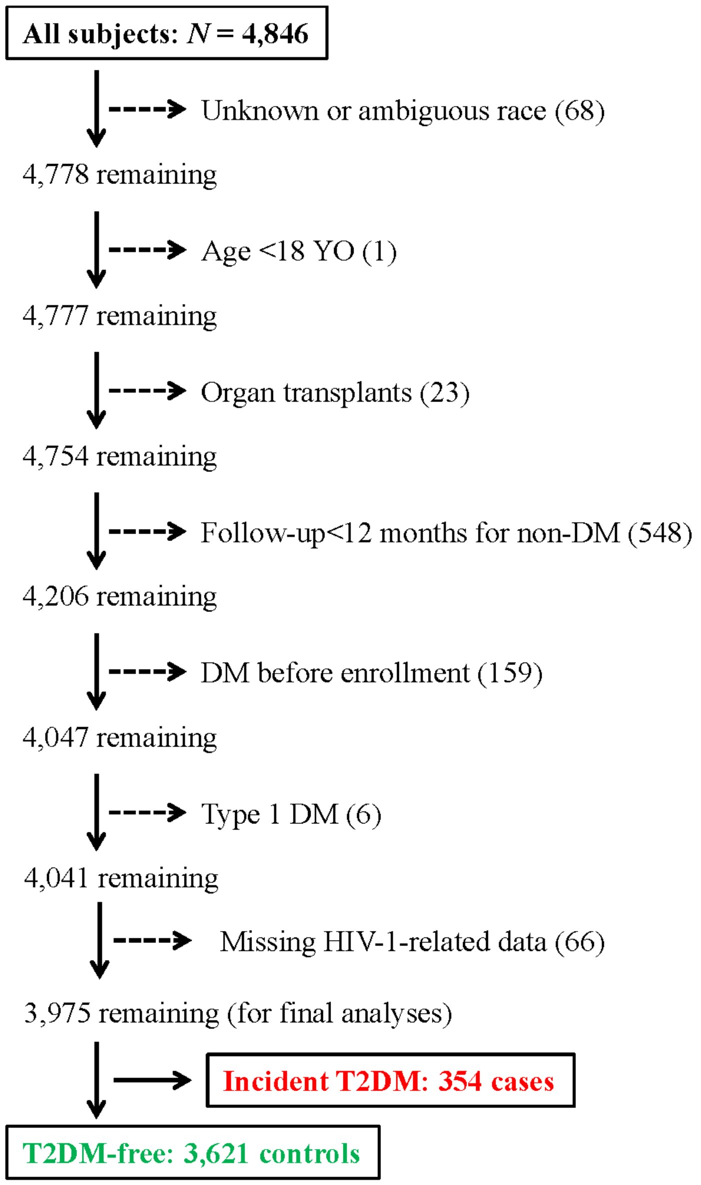

Figure S2. Rates of T2DM per 1,000 person-years (PY) of follow-up, as observed in four enrollment intervals. The 18-year enrollment period (January 1999 to December 2017) is divided into four equal intervals (4.5 years each), and rates of incident T2DM are calculated separately for comparison. Horizontal bars correspond to mean values and the 95% confidence interval for each stratum. AA, African American; EA, European American.

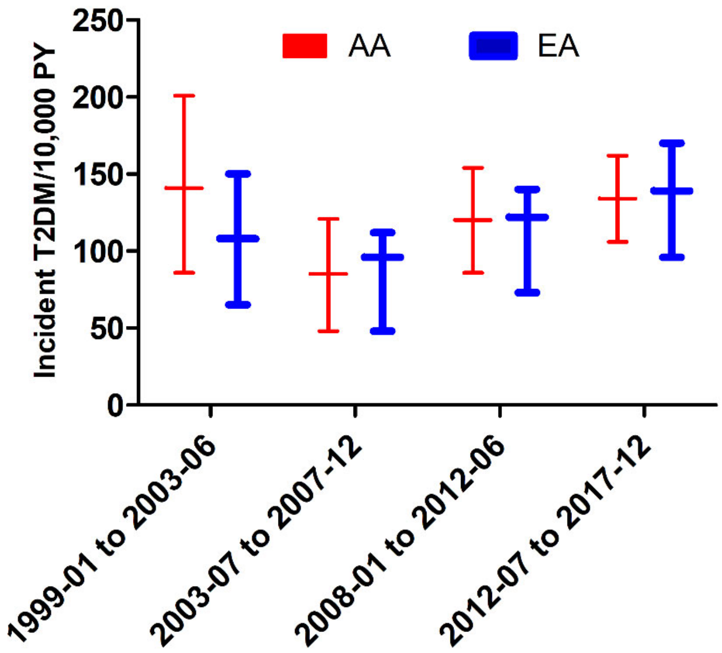

Figure S3. Age of onset for incident type 2 diabetes mellitus (T2DM) as observed in African American (AA) and European American (EA) PLWH. The median age of T2DM diagnosis is 48.9 years in AA and 51.3 years in EA (*P* >0.50).

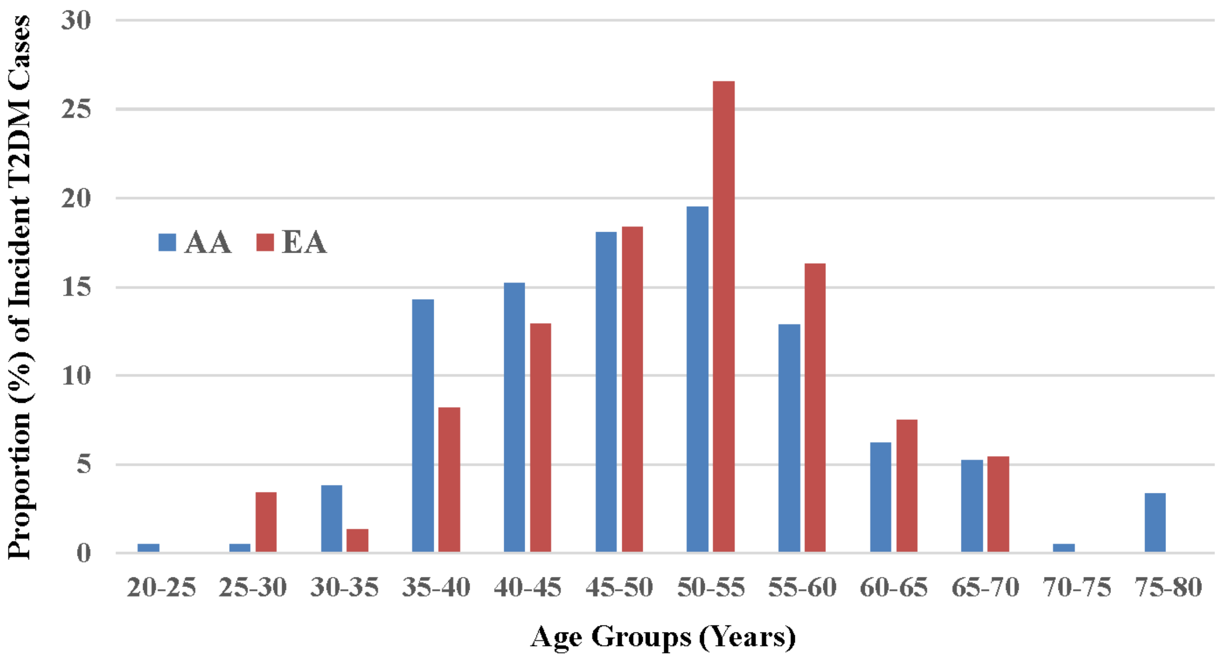

Supplement: Supplementary file 1 [file Presentation_1.pdf]
